# Water Extract of Lotus Leaf Alleviates Dexamethasone-Induced Muscle Atrophy via Regulating Protein Metabolism-Related Pathways in Mice

**DOI:** 10.3390/molecules25204592

**Published:** 2020-10-09

**Authors:** Sang Hee Park, Jieun Oh, Minkyeong Jo, Jin Kyeong Kim, Dong Seon Kim, Han Gyung Kim, Keejung Yoon, Yoonyong Yang, Jeong-ho Geum, Jung-Eun Kim, Su-Young Choi, Ji Hye Kim, Jae Youl Cho

**Affiliations:** 1 Department of Biocosmetics, Sungkyunkwan University, Suwon 16419, Korea; 84701@naver.com; 2 Department of Integrative Biotechnology and Biomedical Institute for Convergence at SKKU (BICS), Sungkyunkwan University, Suwon 16419, Korea; martia96@gmail.com (J.O.); whalsrud1017@naver.com (M.J.); rosekim95@naver.com (J.K.K.); wetdry20@hanmail.net (D.S.K.); hanks523@skku.edu (H.G.K.); keejung@skku.edu (K.Y.); tazemenia@korea.kr (Y.Y.); 3 COSMAX NBT, INC., Seoul 06132, Korea; geumjeongho@cosmaxnbt.com; 4 Biological and Genetic Resources Assessment Division, National Institute of Biological Resources, Incheon 22689, Korea; kimje@cosmaxnbt.com (J.-E.K.); sychoifwp@cosmaxnbt.com (S.-Y.C.)

**Keywords:** *Nelumbo nucifera* Gaertn, leaf of lotus, muscle wasting, dexamethasone-induced muscle atrophy, C2C12 myoblast cells, Atrogin-1, MuRF1, Quercetin 3-*O*-beta-glucuronide

## Abstract

Muscle atrophy is an abnormal condition characterized by loss of skeletal muscle mass and function and is primarily caused by injury, malnutrition, various diseases, and aging. Leaf of lotus (*Nelumbo nucifera* Gaertn), which has been used for medicinal purposes, contains various active ingredients, including polyphenols, and is reported to exert an antioxidant effect. In this study, we investigated the effect of water extract of lotus leaf (LL) on muscle atrophy and the underlying molecular mechanisms of action. Amounts of 100, 200, or 300 mg/kg/day LL were administered to dexamethasone (DEX)-induced muscle atrophy mice for 4 weeks. Micro-computed tomography (CT) analysis revealed that the intake of LL significantly increased calf muscle volume, surface area, and density in DEX-induced muscle atrophy mice. Administration of LL recovered moving distance, grip strength, ATP production, and body weight, which were decreased by DEX. In addition, muscle damage caused by DEX was also improved by LL. LL reduced the protein catabolic pathway by suppressing gene expression of muscle atrophy F-Box (MAFbx; atrogin-1), muscle RING finger 1 (MuRF1), and forkhead box O (FoxO)3a, as well as phosphorylation of AMP-activated kinase (AMPK). The AKT-mammalian target of the rapamycin (mTOR) signal pathway, which is important for muscle protein synthesis, was increased in LL-administered groups. The HPLC analysis and pharmacological test revealed that quercetin 3-*O*-beta-glucuronide (Q3G) is a major active component in LL. Thus, Q3G decreased the gene expression of atrogin-1 and MuRF1 and phosphorylation of AMPK. This compound also increased phosphorylation levels of mTOR and its upstream enzyme AKT in DEX-treated C2C12 cells. We identified that LL improves muscle wasting through regulation of muscle protein metabolism in DEX-induced muscle atrophy mice. Q3G is predicted to be one of the major active phenolic components in LL. Therefore, we propose LL as a supplement or therapeutic agent to prevent or treat muscle wasting, such as sarcopenia.

## 1. Introduction

Muscle atrophy, also known as muscle wasting, refers to a condition in which skeletal muscle mass is lost and is mainly accompanied by impairment of physical activity due to abnormal weakness in muscle strength and function [1]. Muscle atrophy can be caused by various diseases or conditions, such as disuse from illness or injury, malnutrition, diabetes, cachexia associated with certain systemic diseases (e.g., cancer, congestive heart failure, and chronic obstructive pulmonary disease), side effects from pharmaceutical therapy, and aging [2,3]. Reduced muscle mass and function from muscle atrophy increase morbidity and mortality. For example, loss of muscle mass is clinically related to poor prognosis and resistance to pharmaceutical treatment in cancer patients [4]. In addition, loss of muscle mass can result in frequent falls and decreased ability to walk, thereby reducing the quality of life [2]. Therefore, preventing or delaying the onset of muscle atrophy is important. However, an effective treatment for muscle atrophy is lacking [5].

Muscle atrophy is caused by various pathological molecular mechanisms. An imbalanced muscle protein metabolism has been suggested as a pivotal reason for the onset of muscle atrophy [1,2]. Proteins in skeletal muscle are constantly synthesized and degraded. However, muscle loss occurs when the decomposition rate exceeds the synthesis rate. Muscle atrophy F-Box (MAFbx, also known as atrogin-1) and muscle RING finger 1 (MuRF1) have been reported as key regulators in the process of muscle protein breakdown. These enzymes are E3 ubiquitin ligases that are expressed in skeletal muscle and control the degradation of target proteins by promoting polyubiquitination-mediated proteolysis by the 26S proteasome [6,7]. In a rodent model of muscle atrophy, the expressions of atrogin-1 and MuRF1 were highly increased at the early stage of muscle wasting, and high expression persisted throughout the period of accelerated proteolysis. In contrast, knock-out mice without these two proteins showed resistance to denervation-induced muscle atrophy [6,7,8]. These studies clearly demonstrate the importance of atrogin-1 and MuRF1 in muscle atrophy and suggest that these proteins may be targets of muscle atrophy therapy. The gene expressions of atrogin-1 and MuRF1 are regulated by forkhead box O (FoxO), a transcriptional factor that is abundantly present in muscle [9,10]. Activation of FoxO is determined by two mechanisms: cytosol-nuclear shuttling and transcriptional activity [11]. These mechanisms are positively or negatively controlled by phosphorylation, depending on the upstream kinase and phosphor-accepting sites. One of the well-known positive upstream regulators of FoxO is AMP-activated kinase (AMPK), which directly phosphorylates specific sites on FoxO1 to activate its transcriptional function [12,13].

Another major regulator of muscle mass is mammalian target of rapamycin (mTOR) [14]. mTOR, a serine/threonine kinase involved in diverse cellular responses such as cell growth and survival, causes muscle hypertrophy by increasing the rate of muscle protein synthesis [15]. In older animals, the activation of mTOR by exercise has been reported to be blunted [16]. These observations suggest that the mTOR-mediated protein synthesis response to muscle contraction weakens with aging, resulting in muscle atrophy in aged animals. In addition, mTOR has been observed to be decreased in muscle atrophy conditions, such as after rotator cuff tendon rupture in a rat model [17], indicating that mTOR signaling is a key regulator of muscle atrophy across numerous atrophy models. The activity of mTOR is positively regulated by AKT. AKT has been reported to activate mTOR, especially by transducing signals in response to insulin-like growth factor 1 (IGF1), IGF2, and insulin stimulation [18,19].

Lotus (*Nelumbo nucifera* Gaertn), an aquatic plant, is extensively cultivated in Australia, Japan, India, Iran, and China [20]. Traditionally, all parts of lotus, including root, seed, and leaf, have been used for medicinal purposes to treat strangury, skin diseases, diarrhea, fever, gastric ulcers, and hyperlipidemia [21,22]. In addition, previous studies reported that extracts of lotus leaf (LL) containing a number of active ingredients such as alkaloids exhibit antioxidant [23,24], antiviral [25], and antiobesity [26] properties. Several studies have shown that high levels of reactive oxygen species (ROS) can regulate muscle protein metabolism, leading to muscle atrophy [27,28]. Therefore, based on the evidence linking oxidative stress and muscle atrophy, we investigated the efficacy of water extract of LL on dexamethasone (DEX)-induced muscle atrophy in mice in this study.

## 2. Results

### 2.1. LL Administration Has a Positive Effect on Calf Muscle Volume, Surface Area, and Density in DEX-Induced Muscle Atrophy Mice

**Figure 1:**
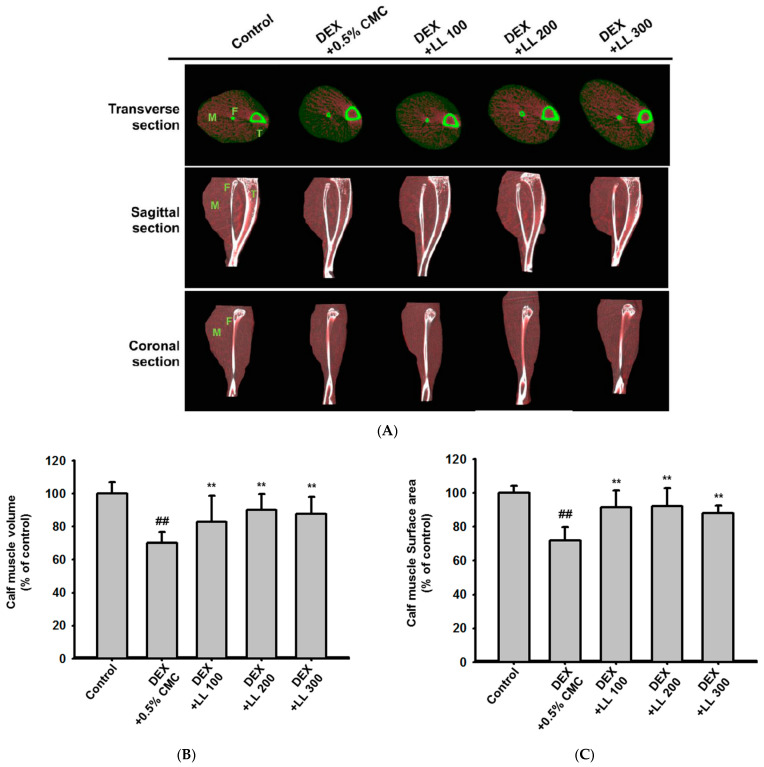

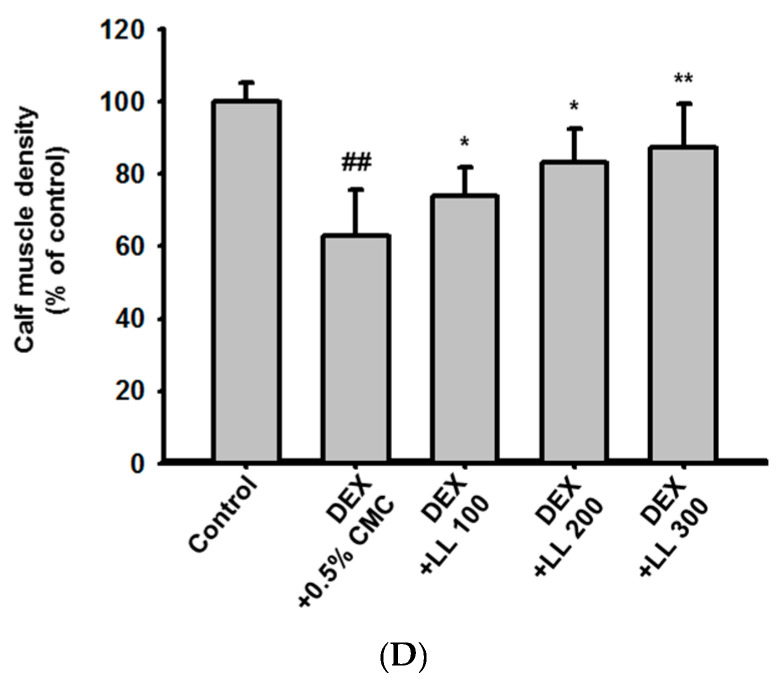
Effect of lotus leaf (LL) on calf muscle properties in dexamethasone (DEX)-induced muscle atrophy mice. (**A**) Micro-computed tomography (CT) images of the calf muscle were acquired at 4 weeks after administration of LL. Images of two-dimensional transverse, sagittal, and coronal slices illustrate the muscle (M) surrounding the fibula (F) and tibia (T). Muscle tissues were identified using boundaries in Hounsfield Units (HU) set to 270 ± 100 and indicated in red. (**B**–**D**) Three-dimensional and two-dimensional reconstructed images were analyzed to evaluate the volume (**B**), surface area (**C**), and density (**D**) of the calf muscle. The data in (**B**), (**C**), and (**D**) are expressed as the means ± SD of an experiment performed with 8 mice per group. ## *p* < 0.01, DEX vs. Control. * *p* < 0.05, ** *p* < 0.01, DEX + LL (100, 200, 300) vs. DEX; DEX, dexamethasone; LL, water extract of lotus leaf; M, muscle; F, fibular; T, tibia.

To evaluate the protective effect of LL against muscle atrophy, we assessed calf muscle properties using micro-CT in DEX-induced muscle atrophy mice. While muscle atrophy was induced in the DEX-administered group, LL (100, 200, and 300 mg/kg) recovered the DEX-induced muscle wasting (Figure 1A). The muscle characteristics were further quantified by considering the muscle distribution in the CT image. The calf muscle volume and surface area were decreased by 30% in the DEX group and remarkably increased in groups treated with LL at all concentrations (100, 200, and 300 mg/kg) compared with the DEX group (Figure 1B and 1C). In addition, calf muscle density, which was reduced by 40% due to DEX, was recovered by 10%, 20%, and 30% in the LL 100, 200, and 300 mg/kg administration groups, respectively (Figure 1D).

### 2.2. Intake of LL Improves the Muscle Function in DEX-Induced Muscle Atrophy Mice

**Figure 2:**
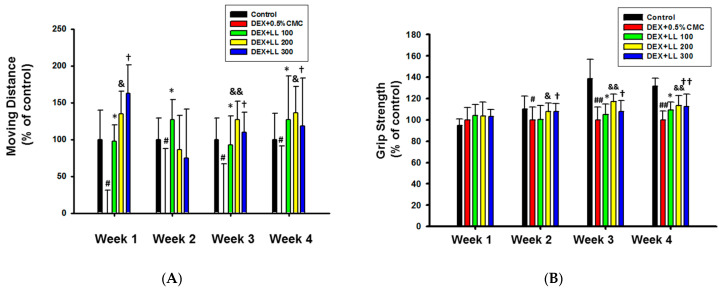

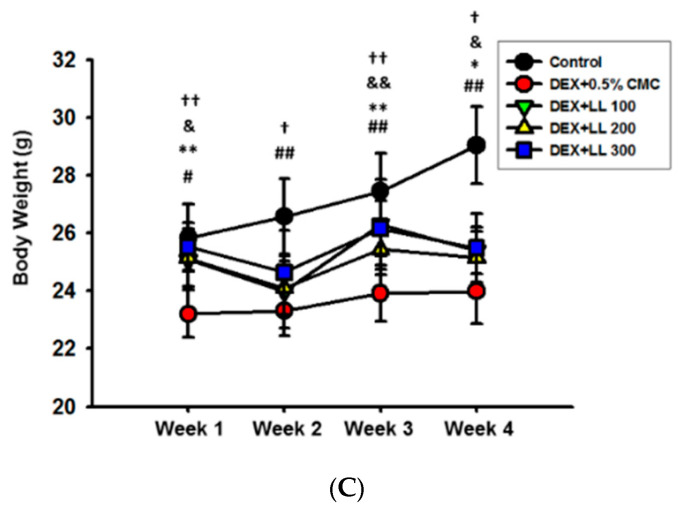
Effect of LL against muscle dysfunction in DEX-induced muscle atrophy mice. (**A**) Grip strength of mice was measured using a grip strength meter. (**B**) Running distance was measured using a treadmill. (**C**) Body weight was measured once a week for 4 weeks. All data are expressed as the means ± SD of an experiment performed with 8 mice per group. # *p* < 0.05, ## *p* < 0.01, DEX vs. Control. * *p* < 0.05, ** *p* < 0.01, DEX + LL 100 vs. DEX. & *p* < 0.05, && *p* < 0.01, DEX + LL 200 vs. DEX. † *p* < 0.05, †† *p* < 0.01, DEX + LL 300 vs. DEX; DEX, dexamethasone; LL, water extract of lotus leaf.

To investigate the effect of LL on muscle function, we measured the exercise capacity of mice. The moving distance was significantly decreased in the DEX group compared with the control group (Figure 2A) However, after 1, 3, and 4 weeks of LL (100, 200, and 300 mg/kg) administration, moving distance was significantly increased compared with the DEX group (Figure 2A). In the second week, only the group treated with 100 mg/kg LL showed significant differences compared with the DEX group, but a trend towards increase was observed with the LL 200 and 300 mg/kg groups. Grip strength of forelimb and hindlimb was also reduced in DEX-administered mice, but recovered by LL treatment starting in the second week (Figure 2B). In addition, loss of body weight was observed in the DEX group, but administration of LL (300 mg/kg) increased body weight throughout the 4 weeks (Figure 2C). LL at 100 and 200 mg/kg also significantly increased body weight at weeks 1, 3, and 4 (Figure 2C).

### 2.3. Oral Administration of LL Prevents DEX-Induced Calf Muscle Damage

**Figure 3:**
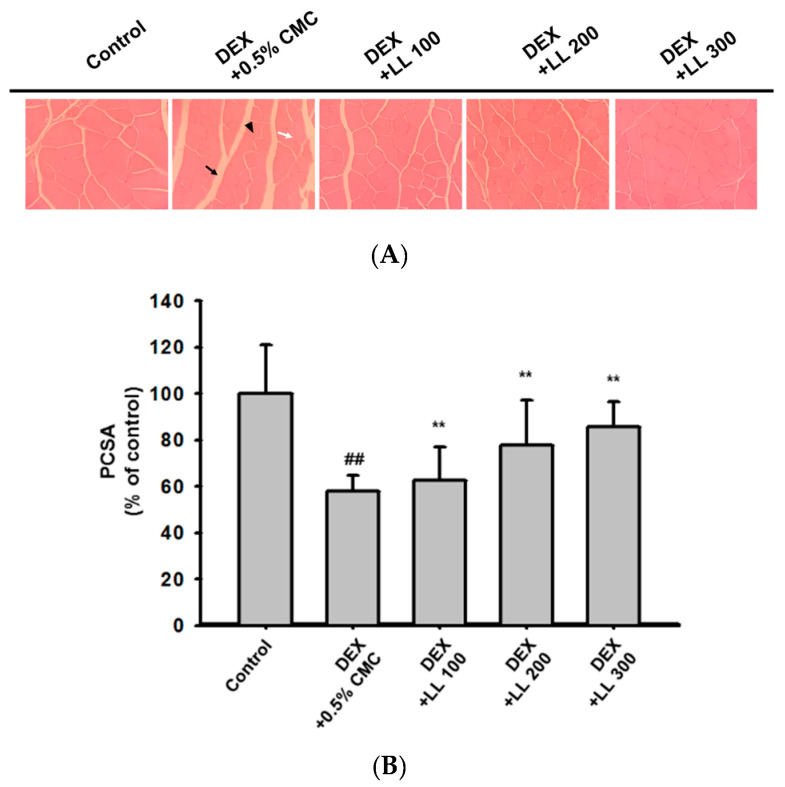
Effect of LL on muscle damage and physiological cross-sectional area (PCSA) of muscle fiber. (**A**) Representative H&E staining of calf muscle from each group. Black arrow, white arrow, and arrowheads indicate the perimysium, the endomysium, and the muscle fiber, respectively. (**B**) Quantification of PCSA of mice calf muscle myofibers. Results are expressed as a relative percentage compared with muscle from the control group. The data in (**B**) are expressed as the means ± SD of an experiment performed with 8 mice per group. ## *p* < 0.01, DEX vs. Control. ** *p* < 0.01, DEX + LL (100, 200, and 300) vs. DEX; DEX, dexamethasone; LL, water extract of lotus leaf.

To explore the effects of LL on muscle damage, we performed histological analysis on the calf muscles. As shown in Figure 3A, muscle fibers of the control group were in intimate contact to form muscle bundles. In contrast, DEX increased perimysium (Figure 3A, black arrow) and endomysium (Figure 3A, white arrow), which are connective tissues surrounding the muscle bundles and muscle fibers. These histological alterations were recovered by intake of LL (100, 200, and 300 mg/kg) (Figure 3A). Moreover, the physiological cross-sectional area (PCSA) in DEX-treated mice was lower than that in the control mice (Figure 3B). Notably, LL (100, 200, and 300 mg/kg) significantly elevated the PCSA compared with mice treated with DEX alone (Figure 3B).

### 2.4. LL Negatively Regulates a Proteolysis-Related Pathway

**Figure 4:**
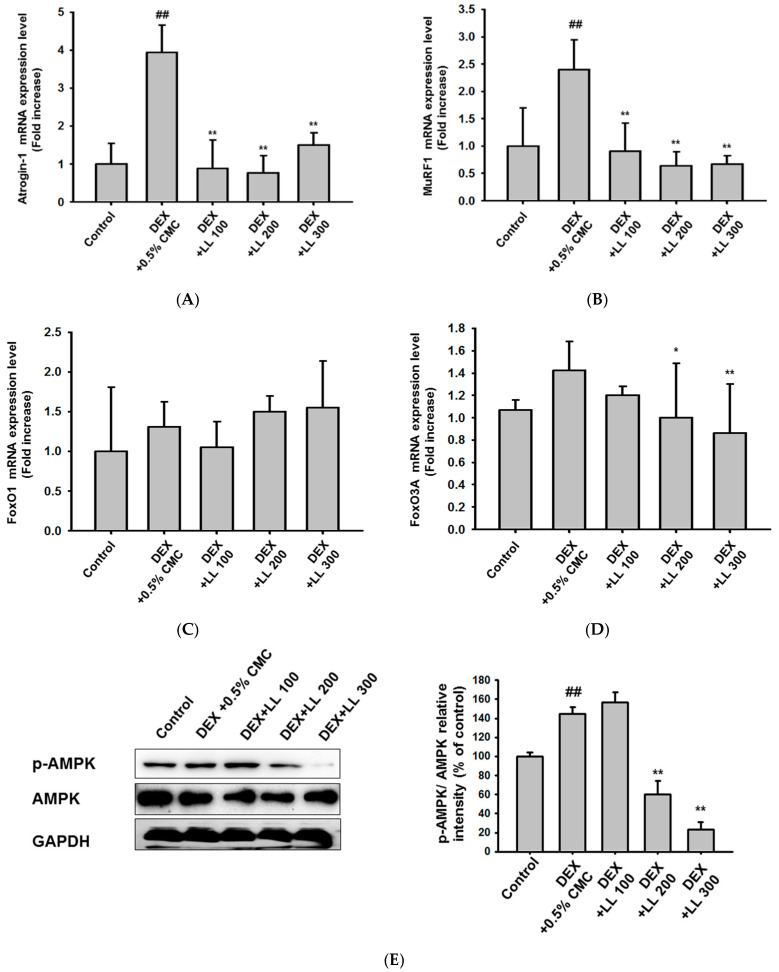
Effect of LL on the muscle protein catabolic pathway. (**A**–**D**) The mRNA expressions of genes encoding atrogin-1 (**A**), MuRF1 (**B**), FoxO1 (**C**), and FoxO3 (**D**) were analyzed by real-time PCR in calf muscle tissue from each group. (**E**) Total and phospho-protein levels of AMPK were analyzed by immunoblotting in calf muscle tissue; GADPH served as control. All data are expressed as the means ± SEM of three independent experiments. Band intensity in (**E**) was measured and quantified using ImageJ. ## *p* < 0.01, DEX vs. Control. * *p* < 0.05, ** *p* < 0.01, DEX + LL (100, 200, and 300) vs. DEX; DEX, dexamethasone; LL, water extract of lotus leaf.

To examine the molecular mechanism by which LL attenuates DEX-induced muscle atrophy, we assessed the levels of proteins that are involved in muscle protein catabolic pathways. DEX administration significantly up-regulated the mRNA expression of genes encoding MuRF1 and atrogin-1, which are essential ubiquitin ligases involved in muscle protein degradation [6,7], within mouse calf muscle (Figure 4A,B). Conversely, LL administration reduced the DEX-mediated increased mRNA levels of these proteins (Figure 4A,B). Because FoxO1 and FoxO3 are key transcription factors that regulate the expression of atrogin-1 and MuRF1 [9,10], we further examined the changes in these proteins in calf muscle tissue of each mouse group. As shown in Figure 4C, DEX and LL did not significantly alter the mRNA expression of genes encoding FoxO1. However, the gene expression of FoxO3a was significantly decreased by administration of LL 200 and 300 mg/kg (Figure 4D). In addition, DEX slightly increased the phosphorylation of AMPK, an upstream kinase of FoxO, and LL significantly reduced the levels of *p*-AMPK without alteration of total proteins at 200 and 300 mg/kg (Figure 4E).

### 2.5. LL Administration Increases the Protein Synthesis-Related Pathway

**Figure 5:**
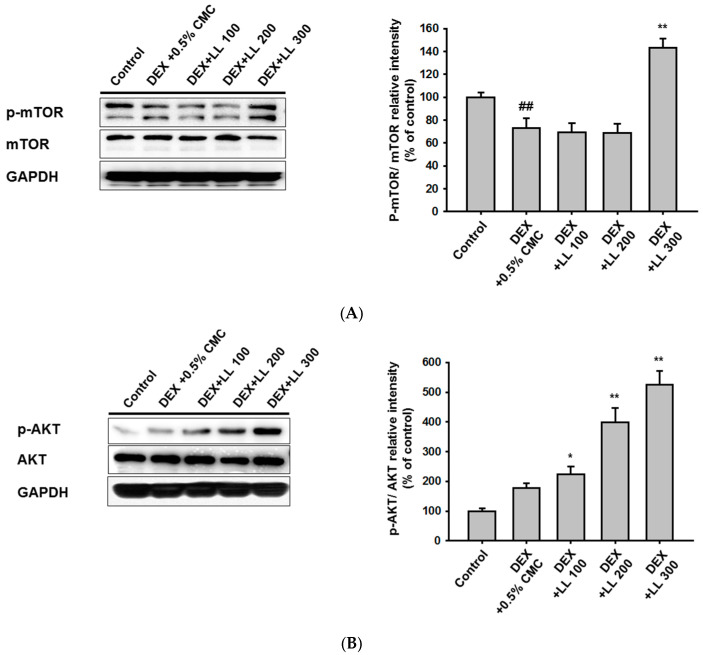
Effect of LL on muscle protein anabolic pathway. (**A**) Total and phospho-protein levels of mTOR were analyzed by immunoblotting in calf muscle tissue from each group; GADPH served as control. (**B**) Total and phospho-protein levels of AKT were analyzed by immunoblotting in calf muscle tissue from each group; GADPH served as control. All data are expressed as the means ± SEM of three independent experiments. Band intensity was measured and quantified using ImageJ. ## *p* < 0.01, DEX vs. Control. * *p* < 0.05, ** *p* < 0.01, DEX + LL (100, 200, and 300) vs. DEX; DEX, dexamethasone; LL, water extract of lotus leaf.

To explore whether LL controls protein synthesis, we investigated the effect of LL on mTOR, a key regulator of the muscle protein anabolic pathway [29]. As shown in Figure 5A, the expression of total mTOR was not influenced by DEX and LL (100, 200, and 300 mg/kg). However, phosphorylation of mTOR was decreased in the DEX group compared with the control group, and administration of LL (300 mg/kg) significantly increased *p*-mTOR levels (Figure 5A). AKT has been reported as a positive regulatory molecule of mTOR, and we thus examined the levels of this kinase [30]. There were no significant changes in *p*-AKT and total AKT in the DEX group compared with the control, but *p*-AKT was considerably increased in the LL (100, 200, and 300 mg/kg) groups (Figure 5B).

### 2.6. LL Contains Q3G as a Major Active Component

**Figure 6:**
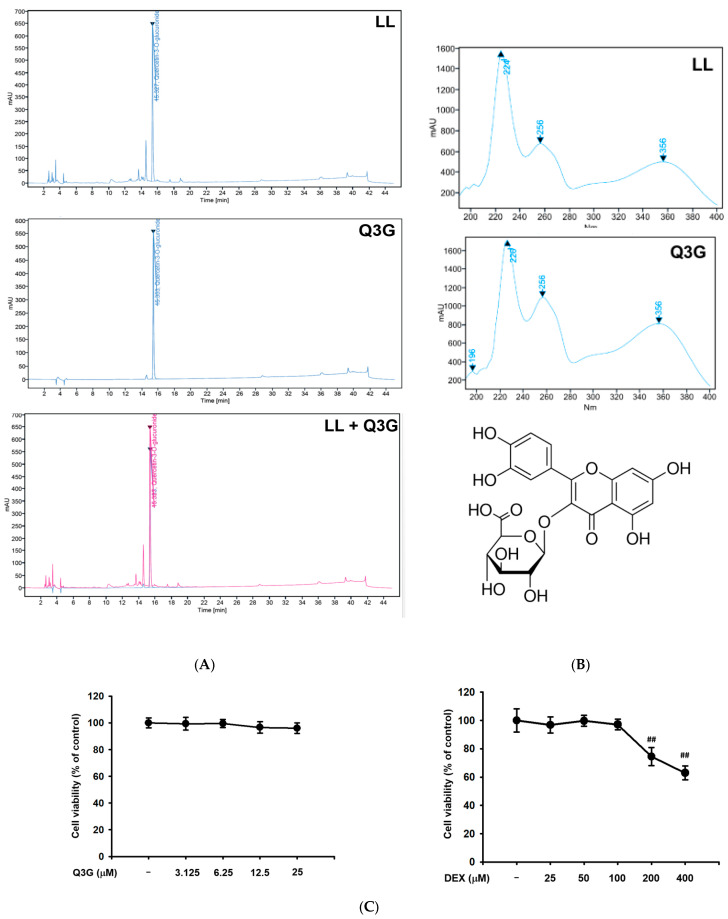

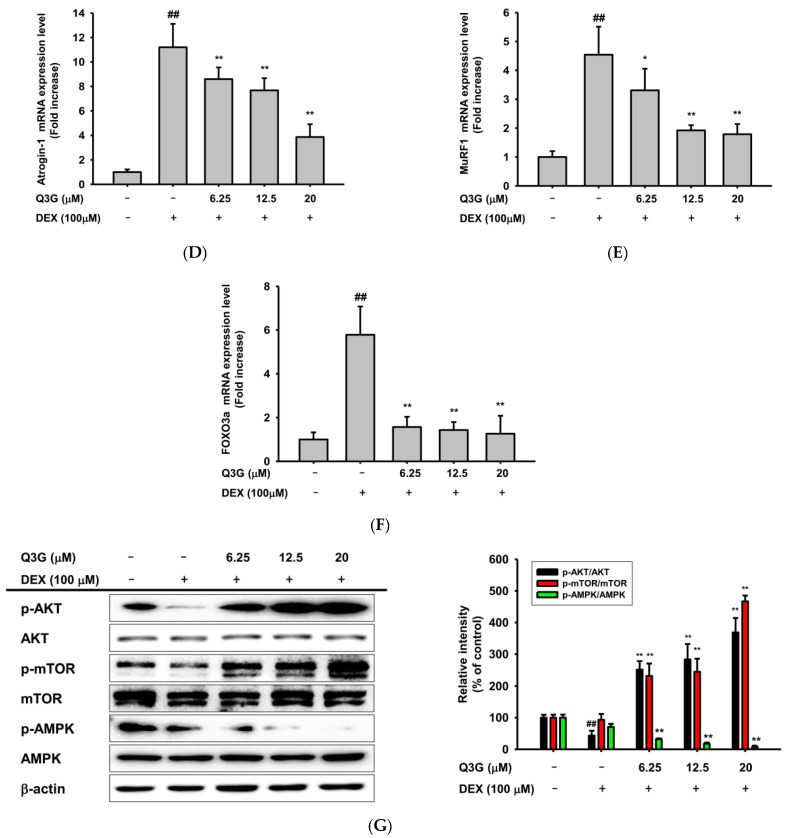
Identification of active phytochemical constituents present in LL. (**A**) HPLC-DAD chromatogram of the LL (top), Q3G (middle), and LL and Q3G (bottom). In the bottom panel, blue and red lines indicate chromatogram of LL and Q3G, respectively. (**B**) The UV spectrum of LL (top) and Q3G (middle) and chemical construction of Q3G (bottom). (**C**) The cytotoxicity of Q3G (3.15, 6.25, 12.5, and 25 μM) (left) and DEX (25, 0, 100, 200, and 400 μM) (right) was examined in C2C12 cells by MTT assay. (**D**–**F**) The mRNA expression level of atrogin-1 (**D**), MuRF1 (**E**), and FoxO3a (**F**) was determined by real-time PCR in DEX-treated C2C12 cells in the presence or absence of Q3G (6.25, 12.5, and 20 μM). (**G**) The phospho- and total forms of AKT, mTOR, and AMPK were detected by immunoblotting assay in DEX-treated C2C12 cells in the presence or absence of Q3G (6.25, 12.5, and 20 μM); β-actin was used as a loading control. The data in (**C**), (**D**), (**E**), (**F**), and (**G**) are expressed as the means ± SEM of three independent experiments. Band intensity in (**G**) was measured and quantified using ImageJ. ## *p* < 0.01, DEX vs. Control. * *p* < 0.05, ** *p* < 0.01, DEX + Q3G (6.25, 12.5, and 20 μM) vs. DEX; LL, water extract of lotus leaf; Q3G, quercetin-3-*O*-beta-glucuronide.

A previous study reported that the flavonoid found in plant compounds primarily provides health benefits [31]. Thus, to determine the active phytochemical composition of LL, we performed HPLC analysis. The highest peak (peak 1) was observed at the retention time (RT) of 15.4 min in LL samples (Figure 6A, top panel). Peak 1 was identified by comparing the RT and UV spectrum with that of the standard form. As shown in Figure 6A,B, RT and UV absorption values of the peak 1 coincide with that of Q3G (Figure 6A,B). The effect of Q3G on muscle protein metabolism-related signaling was further investigated using C2C12 myoblast. MTT assay revealed that Q3G did not affect the viability of C2C12 cells up to a concentrations of 25 μM, whereas DEX showed cytotoxicity at doses exceeding 100 μM (Figure 6C, left and right panels). Based on their cytotoxicity profile, the concentrations of Q3G (6.25, 12.5, and 20 μM) and DEX (100 μM), which were effective but non-cytotoxic, were administered to C2C12 cells. Q3G treatment significantly decreased the DEX-mediated increased mRNA levels of atrogin-1, MuRF1, and FoxO3a at all doses (Figure 6D–F). Phosphorylation levels of AKT and mTOR were decreased by DEX, while those were increased by Q3G at all doses (Figure 6G). On the other hand, Q3G dose-dependently suppressed phosphorylation of AMPK (Figure 6G). These results are consistent with the experimental finding obtained from LL-administered mice, which suggests that Q3G is one of the major active phenolic components of LL.

## 3. Discussion

Muscle atrophy is a destructive symptom that occurs in cachexia as well as aging. Despite the prevalence of muscle wasting, most treatments depend on exercise or protein supplementation, and only few drugs are available in clinical practice for muscle atrophy conditions [32]. In 1993, megestrol acetate (MA) was approved by the US Food and Drug Administration as a treatment for cachexia in cancer and acquired immune deficiency syndrome (AIDS) patients [33]. However, administration of MA is accompanied by side effects such as thromboembolism, temporary adrenal insufficiency, and central nervous system damage [32]. In severe cases, anabolic steroids such as methandrostenolone are also used, but those are limited due to side effects [34]. Thus, developing effective treatments or supplements with few side effects for muscular atrophy is important. In this study, we reported the preventive effect of LL on muscle dysfunction using the DEX-induced muscle atrophy mouse model. In addition, the molecular mechanisms were clearly identified through molecular biological analysis using tissue samples of each group.

Prior to the evaluation of effects on muscle atrophy, we administered various doses of LL (100, 200, and 300 mg/kg) and monitored body weight of mice for 4 weeks. All mice showed normal increment in body weight and did not show any toxic signs such as diarrhea and sleep throughout the treatment (data not shown). However, body weight gain was slightly increased in the LL (100, 200, and 300) groups compared to the control group (data not shown). These results indicate that LL has no toxicity up to 300 mg/kg. Interestingly, administration of LL significantly increased the body weight of mice in the condition in which muscle dysfunction was induced by DEX (Figure 2C). In addition, administration of LL (100, 200, and 300 mg/kg) increased the moving distance and grip strength (Figure 2A,B), implying that LL improves the DEX-induced impairment of muscle functions. To confirm that this improvement was due to muscle strengthening, we also examined changes in muscle properties using micro-CT analysis. In parallel with results on muscle function, oral administration of LL (100, 200, and 300 mg/kg) also increased calf muscle volume, surface area, and density (Figure 1B–D). Histological analysis of calf tissue also revealed that LL (100, 200, and 300 mg/kg) restored DEX-induced muscle tissue atrophy (Figure 3A,B). These results clearly indicate that LL has a preventive effect against DEX-induced muscle atrophy.

Various cellular and molecular studies have been conducted to examine the molecular mechanisms that contribute to the onset of muscle atrophy. The breakdown of muscle proteins by atrogin-1 and MuRF1 has been identified as the main cause of muscle wasting, and these proteins are considered as targets for therapeutic agents [8]. In addition, previous studies reported that DEX, a synthetic glucocorticoid, promotes atrogin-1 and MuRF1-medated proteolysis, leading to muscle wasting [6,35]. Thus, we investigated whether LL affected atrogin-1 and MuRF1 in calf tissues. As previously reported [35], DEX increased the mRNA expressions of atrogin-1 and MuRF1. Notably, our results showed that LL administration diminished the expression of these genes at all concentrations (Figure 4A,B). In addition, mRNA expression of FoxO3a and the level of *p*-AMPK were reduced by LL treatment at doses of 200 and 300 mg/kg (Figure 4D,E). Considering that AMPK positively regulates the transactivation of FoxO1 and FoxO3 [12,13,36], we speculated that the down-regulation of MuRF1 and atrogin-1 by LL may be from inhibition of activity and expression of FoxO. Interestingly, LL significantly reduced the gene expression of atrogin-1 and MuRF1 without alteration in FoxO expression and *p*-AMPK levels at concentration of 100 mg/kg (Figure 4E). This suggests that the gene expression of atrogin-1 and MuRF is regulated by multiple molecules.

We also observed that LL up-regulated *p*-mTOR levels by activating AKT at a dose of 300 mg/kg (Figure 5A,B). AKT/mTOR signaling is critical for protein synthesis at transcriptional and translational levels [37]. Rapamycin-sensitive mTOR (also termed as mTORC1) is essential for hypertrophy induced by resistance exercise (RE) [38]. In addition, activation of AKT/mTOR signaling has been known to prevent muscle atrophy in vivo [30]. Indeed, nutrient supplementation, such as with leucine [39], targets the activation of this signaling to improve muscle atrophy symptoms. Therefore, we speculated that activation of the AKT-mTOR pathway contributed to the increase in calf muscle mass and function by LL. However, LL did not show a significant effect on mTOR at 100 and 200 mg/kg (Figure 5A). This suggests that the primary mechanism for alleviating muscle atrophy by LL administration is inhibiting proteolysis via atrogin-1 and MuRF1.

HPLC analysis showed that LL contains a large amount of Q3G (Figure 6). Q3G is a major quercetin metabolite observed in plasma after quercetin intake and a glycoside derivative of quercetin [40]. Quercetin is a flavonoid widely distributed in functional foods, including vegetables and fruits, and has powerful antioxidant properties [41]. Oxidative damage caused by free radicals has been linked to a number of diseases, including cancer, cardiovascular and inflammatory diseases, and aging [42], and quercetin exhibits therapeutic efficacy for these degenerative diseases based on its strong antioxidant activity. In addition, previous studies reported that quercetin prevents disused muscle atrophy by suppressing ubiquitin ligase or protecting mitochondria in tail-suspension mice and denervated mice, respectively [43,44]. Q3G (Figure 6B bottom panel), like quercetin, has antitumor [45,46], *anti*-inflammatory [47,48], and antiviral activities [49], as well as antioxidant properties [50]. In addition, quercetin glycosides have been reported to attenuate DEX-induced muscle atrophy in mice [51]. Indeed, in this study, Q3G suppressed the gene expression of atrogin-1 and MuRF1 and phosphorylation of AMPK in DEX-treated C2C12 cells, as well as up-regulated phosphorylation of mTOR and AKT (Figure 6D–G). Furthermore, a pharmacokinetic comparison study revealed that the area under the curve (AUC) of Q3G was 18-fold higher than that of quercetin in rat plasma after oral administration, indicating that Q3G was superior to quercetin in terms of absorption rate [52]. Based on these previous reports and our results, we predict that Q3G is the active component in LL for alleviating DEX-induced muscle atrophy.

## 4. Materials and Methods

### 4.1. Materials

Dexamethasone, (3-4,5-dimethylthiazol-2-yl)-2,5-diphenyltetrazolium bromide (MTT), Q3G, and sodium dodecyl sulfate (SDS) were purchased from Sigma Chemical Co. (St. Louis, MO, USA). Dulbecco’s Modified Eagle’s medium (DMEM) and Roswell Park Memorial Institute (RPMI) 1640, Fetal bovine serum (FBS), phosphate-buffered saline (PBS), and TRIzol reagent were purchased from GIBCO (Grand Island, NY, USA). Antibodies specific for phosphorylated or total forms of signaling proteins were purchased from Cell Signaling Technology (Danvers, MA, USA).

### 4.2. Preparation of LL

Dried LL was obtained from COSMAX NBT INC. (Seoul, South Korea). We extracted 85 kg of LL using water at 95 °C for 4 h. The solution was filtrated and concentrated. Dextrin was added (dextrin:dry matter content = 3:7), and the sample was dried with a yield of 22.6% (extract powder g/raw material g).

### 4.3. Animal Experiment

Institute of Cancer Research (ICR) mice (male, 8 weeks old, 24.25 ± 1.06 g) were purchased from Orient Bio (Seongnam, Korea). Mice were randomly designated into five groups as follows: (I) Control, (II) DEX and 0.5% (CMC)-treated group (DEX + 0.5% CMC), (III) DEX and 100 mg/kg/day LL-treated group (DEX + LL 100), (IV) DEX and 200 mg/kg/day LL-treated group (DEX + LL 200), and (V) DEX and 300 mg/kg/day LL-treated group (DEX + LL 300) (*n* = 10 per group). The animals were housed in polycarbonate cages with wood pulp bedding, which was changed twice a week. Mice were fed a pellet rodent diet (Samyang, Daejeon, Korea) and tap water. The facility was maintained under specific pathogen-free conditions at a temperature of 23 ± 2 °C and a humidity of 55 ± 10%, with a 12-h light/dark cycle. After a 1-week acclimation, tap water was provided for the normal group, while other groups (control and drug-testing groups) were provided with 0.01% *w/v* DEX in the drinking water for 4 weeks (day 1 to day 28). Each water bottle was replaced daily. LL (100, 200, or 300 mg/kg) dissolved in 0.5% CMC solution was orally administered daily for 4 weeks, and the 0.5% CMC solution was orally administered to the normal group and DEX + 0.5% CMC group. Once a week, exercise endurance and grip strength were tested, and the body weight of each mouse was recorded before every forced treadmill exercise. At the end of the experiment, mice were sacrificed under anesthesia with ether. The gastrocnemius muscle was isolated and stored at −70 °C for Western blotting, gene expression analysis, and histological analysis. Blood serum was also prepared for ATP production assay. All studies were performed according to the guidelines established by the Sungkyunkwan University Institutional Animal Care and Use Committee (Suwon, Korea; approval ID: SKKUIACUC2018-10-16-1).

### 4.4. Cell Culture

Murine myoblast C2C12 cell line was purchased from the American Type Culture Collection (ATCC, Manassas, VA, USA). The C2C12 cells were cultured with 10% heat-inactivated FBS, glutamine, and antibiotics (penicillin and streptomycin) at 37 °C under 5% CO_2_. For induction of myogenic differentiation, C2C12 myoblasts were transferred to differentiation medium of DMEM supplemented with 2% Horse Serum (16050130, Thermo Fisher scientific, Waltham, MA, USA) and were cultured for 7 days. The medium was changed with fresh differentiation medium every 2 days.

For mRNA analysis and Western blotting, differentiated C2C12 cells were seeded in 6 well or 10 cm plates, and cultured overnight. Then, C2C12 cells were treated with quercetin 3-*O*-beta-glucuronide (Q3G) (6.25, 12.5 20 μM) and DEX (100 μM) for 24 h.

### 4.5. Micro-Computed Tomography (CT) Imaging

Muscle volume and density were measured by a high-resolution micro-CT scanner (SkyScan 1076, SkyScan, Kontich, Belgium) at the Center for University-Wide Research Facilities at Jeonbuk National University. Gastrocnemius muscles dissected from mice were scanned in 360° in vertical rotation steps of 0.6° with a desktop micro-CT unit at 5-mm resolution. Muscle tissues were identified using boundaries in Hounsfield Units (HU) set to 270 ± 100. The scanned images were reconstructed in 3D images and analyzed with CTVox software (version 3.0, Skyscan, Kontich, Belgium). A cross-sectional area of each muscle group was analyzed with CTAn software (version 1.18).

### 4.6. Running Test

Running distance was measured using a treadmill (Panlab, Barcelona, Spain) once a week for 4 weeks. The running was started at 7 m/min on a 15° incline, and the speed was gradually increased by 3 m/min every 3 min until it reached 30 m/min and then was maintained. The mice ran for at least 20 min, and the test was stopped when the mice were exhausted.

### 4.7. Grip Strength Test

A grip strength meter (Panlab, Barcelona, Spain) was utilized to measure the grip strength of mice. After setting the gauge to 0 g, each mouse was allowed to grasp the pull grid; the mouse’s tail was then slowly pulled back until the mouse missed the pull grid. Three consecutive tests were performed for each mouse, and the mean value was calculated.

### 4.8. Histology

After 4 weeks of LL administration, the calf muscles were removed and fixed in 10% neutral buffered formalin. The fixed muscle tissue was embedded in paraffin, and the paraffin blocks were cut into 4 μm thick sections. To assess tissue morphology, transverse sections of muscle tissues were stained with hematoxylin (ab220365 Abcam, Cambridge, UK) and eosin (H&E). The sections were examined under a microscope (Eclipse TE 2000-U; Nikon, Düsseldorf, Germany). The PCSAs were analyzed using ImageJ software (version 2020).

### 4.9. Real-Time Polymerase Chain Reaction (PCR)

Total RNA was isolated from the calf muscle or C2C12 cells using TRIzol reagent according to the manufacturer’s instructions. Complementary DNA (cDNA) was synthesized from 1 μg total RNA using a cDNA synthesis kit. Real-time PCR was conducted using the qPCRBIO SyGreen Blue Mix Lo-ROX (PCR Biosystems Ltd., London, UK) according to the manufacturer’s instructions. The PCR reaction was performed using a real-time C1000 thermal cycler (Bio-Rad Laboratories Inc., Hercules, CA, USA) under the following conditions: 10 s denaturation time at 95 °C, 10 s annealing time at 58 °C, and 60 s extension time at 72 °C, for 39 cycles. The gene expression of *atrogin-1*, *MuRF1*, *FoxO1*, and *FoxO3a* was normalized to *GAPDH* and expressed as fold increase (normal level was set as 1). The sequences of the primers used in this study are shown in Table 1.

### 4.10. Western Blotting Analysis

Total lysates of calf muscle or C2C12 cells were prepared by homogenization in cold lysis buffer (20 mM Tris-HCl, pH 7.4; 2 mM EDTA; 2 mM ethyleneglycotetraacetic acid (EGTA); 1 mM DTT; 50 mM β-glycerol phosphate; 0.1 mM sodium vanadate; 1.6 mM pervanadate; 1% Triton X-100; 10% glycerol; 10 μg/mL aprotinin; 10 μg/mL pepstatin; 1 μM benzamide; and 2 μM PMSF). The protein lysates were clarified by centrifugation at 12,000 rpm for 5 min at 4 °C, and the supernatants were used for Western blotting analysis [53]. Protein samples were separated on 10% SDS-polyacrylamide gels and transferred by electroblotting onto a polyvinylidene difluoride membrane (Merck Millipore, Billerica, MA, USA). The membrane was probed with following primary antibodies overnight at 4 °C: *anti*-mTOR (CST#2972, Cell Signaling Technology, Boston, MA, USA), *anti*-*p*-mTOR (CST#2971, Cell Signaling Technology), *anti*-*p*-AKT (CST#4058, Cell Signaling Technology), *anti*-AKT (CST#9272, Cell Signaling Technology), *anti*-AMPK (CST#2532, Cell Signaling Technology), *anti*-*p*-AMPK (CST#2535, Cell Signaling Technology), *anti*-AMPK (CST#2532, Cell Signaling Technology), *anti*-β-actin (CST#4967, Cell Signaling Technology), and *anti*-GAPDH (SC-166545, Santa Cruz Biotechology, CA, USA). The membrane was washed three times with Tris-buffered saline with Tween 20 (TBST) and then incubated with a secondary antibody conjugated with horseradish peroxidase in BSA for 1 h. Immunoreactive bands were detected with the ChemiDoc Imaging System (Luminograph III, ATTO, Amherst, NY, USA). Band intensity was measured and quantified using ImageJ [54].

### 4.11. High-Performance Liquid Chromatography (HPLC) Analysis

HPLC analysis was performed using an Agilent infinity 1260 II equipped with an autosampler, a quaternary pump, column temperature control, and DAD detector. To prepare a working solution, 40–45 mg LL powder and 2 mg Q3G were each dissolved in 10 mL MeOH (Honeywell Burdick & Jackson, NJ, USA). Each solution was then filtered using a 0.45 μm membrane filter and injected into the HPLC system. LL and Q3G samples were analyzed using a C18 column with an internal diameter of 4.6 × 250 mm (Phenomenex, Torrance, CA, USA). The detector wavelength was 255 nm and injection volume was 10 μL. The mobile phase consisted of solvent A (1% acetic acid in water) and solvent B (1% acetic acid in acetonitrile). The flow was 0.8 mL/min and the column temperature was 40 °C. The gradient elution conditions were as follows: 10% B for 0–5 min; 10–30% B for 5–10 min; 30–100% B for 10–35 min; 100% B for 35–37 min and 100–10% B for 37–40 min; and 10% B for 40–45 min [55].

### 4.12. MTT Assay

C2C12 cells were treated with Q3G (3.15, 6.25, 12.5, and 25 μM) or DEX (25, 50, 100, 200, and 400 μM) for 24 h. Then, 10 μL MTT solution was added and incubated for 3 h, and the reaction was stopped by stopping solution (15% SDS), as reported previously [56]. The samples were then incubated overnight, and the absorbance of MTT formazan was measured at a wavelength of 540 nm.

### 4.13. Statistical Analysis

All experiments were performed with 10 mice per group. Data in Figure 1, Figure 2 and Figure 3 are expressed as the means ± standard deviation (SD) calculated from at least eight mice. For Western blot analysis, real-time PCR, and MTT assay in Figure 4, Figure 5 and Figure 6 (C–G), three independent experiments were performed. Analysis of variance (ANOVA) with the Mann-Whitney U test was used for statistical comparison. For all analyses, *p* < 0.05 was considered statistically significant.

## 5. Conclusions

**Figure 7:**
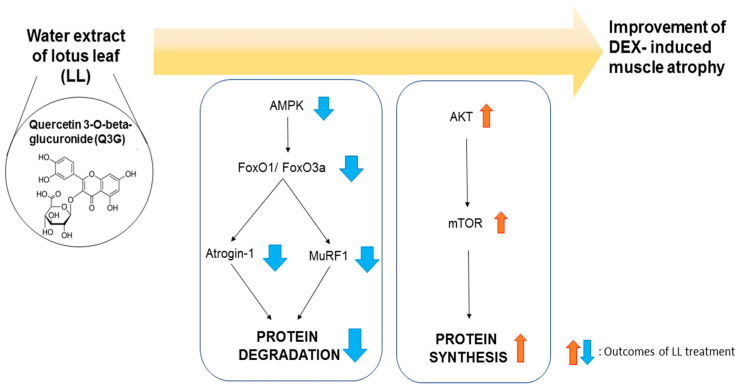
Schematic diagram of the preventive effects of LL on dexamethasone-induced muscle atrophy.

In conclusion, this study provides evidence for the efficacy of LL in preventing muscle atrophy and reveals the underlying molecular mechanisms. Administration of LL for 4 weeks significantly increased muscle mass and muscle function that was inhibited by DEX in mice. These results indicate that LL has the ability to prevent muscle wasting. LL activated AKT in calf muscle, but suppressed AMPK. As a result, the activity of FoxO1/3 and mRNA expression of atrogin-1 and MuRF1, proteins involved in the pivotal pathway of proteolysis, were significantly reduced by LL. In addition, mTOR, a central molecule for protein synthesis, was positively regulated by LL (Figure 7). LL contains a large amount of Q3G, which is expected to be an active ingredient in attenuating muscle atrophy. Based on these results, LL is recommended to be used as a supplement or therapeutic agent to prevent or treat muscle atrophy, including sarcopenia.

## Figures and Tables

**Table 1:** Primer sequences used in this study.

Target		Sequences (5′ → 3′)
*Atrogin-1*	Forward	TTCAGCAGCCTGAACTACGA
	Reverse	AGTATCCATGGCGCTCCTTC
*MuRF1*	Forward	GAGGGGCTACCTTCCTCTCA
	Reverse	AGAGGAACGCTGCCTTTCAA
*FoxO1*	Forward	AGTGGATGGTGAAGAGCGTG
	Reverse	GAAGGGACAGATTGTGGCGA
*FoxO3A*	Forward	GAACCTGTCCTATGCCGACC
	Reverse	TGGGACAAAGTGAGCCGTTT
*GAPDH*	Forward	GGTTGTCTCCTGCGACTTCA
	Reverse	CATTGAGAGCAATGCCAGCC
